# Photocatalytic degradation of 2,4-dichlorophenol using nanomaterials silver halide catalysts

**DOI:** 10.1007/s11356-024-31921-1

**Published:** 2024-01-15

**Authors:** Mahlako Mary Moja, António Benjamim Mapossa, Evans Martin Nkhalambayausi Chirwa, Shepherd Tichapondwa

**Affiliations:** 1 Department of Chemical Engineering, University of Pretoria, Pretoria, 0002 South Africa; 2 Department of Chemical and Petroleum Engineering, University of Calgary, 2500 University Drive NW, Calgary, AB T2N 1N4 Canada

**Keywords:** Advanced oxidation processes, Photocatalysts, Visible light, Wastewater treatment

## Abstract

In this study, the photocatalytic activity of nanomaterials Ag/AgX (X = Cl, Br, I) is reported. Highly efficient silver halide (Ag/AgX where X = Cl, Br, I) photocatalysts were synthesized through a hydrothermal method. The samples were characterized using a range of techniques such as X-ray diffraction (XRD), scanning electron microscopy (SEM), and Brunauer–Emmett–Teller (BET) to check their structural, morphology, textural and optical properties. In addition, the photocatalytic activity of photocatalysts was evaluated through the degradation of 2,4-dichlorophenol (2,4-DCP) under UV and visible light irradiation. XRD analysis confirmed the presence of a single-phase structure (pure phase) in the synthesized photocatalysts. SEM micrographs showed agglomeration with a non-uniform distribution of particles, which is a characteristic of surfactant-free precipitation reactions in aqueous media. The Ag/AgBr photocatalyst exhibited the best degradation efficiency, resulting in 83.37% and 89.39% photodegradation after 5 h of UV and visible light irradiation, respectively. The effect of catalyst loading, initial solution pH, and 2,4-DCP concentration was investigated for the best-performing Ag/AgBr photocatalyst. The degradation kinetics were best described by the pseudo-first-order Langmuir–Hinshelwood model. The photocatalytic capacity of Ag/AgBr decreased by 50% after five reuse cycles. SEM images revealed heightened levels of photodegradation on the catalyst surface. The study proved the feasibility of using simple synthesis methods to produce visible light active photocatalysts capable of degrading refractory phenolic pollutants in aqueous systems.

**Supplementary Information:**

The online version contains supplementary material available at 10.1007/s11356-024-31921-1.

## Introduction

Water pollution is a major global issue, which presents a major risk to the long-term sustainability of the environment and its ability to support human life (Schwarzenbach et al. 2010; Kurniawan et al. 2012). The rapid increase in the human population has led to a corresponding increase in industrial production to satisfy daily needs such as food and energy. As a consequence, water bodies across the world are receiving higher volumes of effluent containing a wide range of priority and emerging pollutants, most of which are constituted from aromatic organic compounds and their halogenated congeners as well as heavy metals (i.e. Cu, Sb, Pu) (Ullah et al. 2013; Fechete et al. 2012; Reddy et al. 2016; Zhang et al. 2020). The presence of these compounds in aqueous systems presents a major problem, due to their intrusiveness and ability to interfere with the normal functioning of biological processes. For example, these compounds disrupt metabolic processes (Giulivo et al. 2016) and endocrine systems in mammals (Marty et al. 2018; Choi et al. 2004).

Phenolic compounds are harmful to human health, as they can cause digestive problems, and liver and kidney damage (Anku et al. 2017). The presence of phenolic compounds and their derivatives in drinking water at concentrations as low as 1 mg L^-1^ may cause serious public health issues, death of aquatic life, and loss of biodiversity (Prado et al. 2002). Phenolic compounds tend to photodegrade into several by-products, some of which have higher toxicities than the parent compounds. Particularly, the chlorinated organic compound 2,4-dichlorophenol (2,4-DCP) is the photodegradation by-product of triclosan (TC), a common antimicrobial agent as well as 2,4-dichlorophenoxyacetic acid (2,4-D) herbicide (Melián et al. 2013). To reduce the risk of 2,4-dichlorophenol in the environment, remediation through the development of suitable alternative removal techniques is needed. Several technologies such as adsorption (Melián et al. 2013), ozonation (Aziz et al. 2018), biological treatment (Guo et al. 2023), and electrochemical treatment (Liu et al. 2019) have been investigated with varying success. However, these technologies have disadvantages such as high operational cost, low efficiency, and the production of excessive by-products and secondary waste. Photocatalysis has been identified as an economically feasible and environmentally benign technology that is capable of degrading and effectively mineralizing a wide range of organic compounds including tetracycline (Tang et al. 2023), recalcitrant phenolics (Adenuga et al. 2019). TiO_2_ and ZnO are among the most widely investigated photocatalysts as they are inexpensive, non-toxic and structurally stable (An et al. 2016; Kaneco et al. 2006; Xiao et al. 2015). However, their large band gaps (> 3.2 eV) limit their application to activation by ultraviolet light irradiation (Cui et al. 2015; Meng and Zhang 2016; Bhatt and Patel 2019). Example, Jia et al. (2022) reported that among the many forms, the TiO_2_ nanotubes (TiO_2_-NTs) structure with corrosion resistance and large specific surface area, is considered as a matrix for photo-electrocatalysis due to the geometric, electrical and optical properties.

Plasmonic photocatalysts which typically consist of noble metals (Au, Ag, Pt, Cu), display high adsorption coefficients in the UV–visible-near infrared spectral range, due to their strong surface plasmon resonance (SPR) properties (Cui et al. 2015; Ye et al. 2012). Silver nanoparticles exhibit efficient plasmon resonance in the visible region. Silver halides are highly photosensitive and can be utilized as metallic silver precursors. AgX absorbs visible light photons to generate electron–hole pairs, which in turn generate highly reactive free radicals which non-selectively, degrade organic pollutants (Rehan et al. 2018). Since the pioneering work of Huang et al. (2008), silver-halide-based plasmonic photocatalysts (denoted Ag/AgX; X = Cl, Br) have attracted extensive interest from researchers owing to their superior photocatalytic performance for degradation of organic contaminants (Wang et al. 2008; Jiao et al. 2019; Wang et al. 2009; Zhu et al. 2015) and inactivation of bacteria (Tian et al. 2014; Hu et al. 2010). Regarding the influence of different halogen ions on the photocatalytic activity of Ag/AgX, Huang et al. found that Ag@AgBr exhibited higher activity than Ag@AgCl for degradation of methyl orange (Wang et al. 2009). Furthermore, uniform cubic Ag@AgCl and Ag@AgBr plasmonic photocatalysts were synthesized by a facile green route (Li et al. 2018). The photocatalytic activities of Ag@AgCl and Ag@AgBr were compared using degradation of methyl orange (MO) dye under visible-light irradiation. The authors reported that Ag@AgBr showed higher photocatalytic activity for MO degradation.

However, previous studies have rarely compared the influence of these catalysts on the photocatalytic activity for organic compounds under different sources simultaneously (i.e., UV and visible light irradiation, which is therefore endeavoured here. Furthermore, these studies have focused only on the influence of these photocatalysts for dye degradation. To the best of our knowledge, the study where the authors explore a simple hydrothermal method to synthesize the Ag/AgX (X = Cl, Br, I) heterogeneous photocatalysts and subsequently apply them in 2,4-dichlorophenol degradation under two sources simultaneously UV and visible light irradiation has not yet been explored. The structural, morphological, optical and photoelectrochemical properties were determined. The optimum operating conditions, kinetic study, and recyclability were conducted on for the best-performing silver halide photocatalyst. Additionally, a degradation mechanism was also proposed.

## Materials and methods

### Materials

Chemicals such as p-benzoquinone (CAS No. 106–51-4) (98% purity), isopropanol (CAS No. 67–63-0) (99.5% purity), and 2,4-dichlorophenol (CAS No. 120–83-2) (≥ 98.5% purity) were supplied by Sigma-Aldrich supplied. Acetic acid (glacial) AR (CAS No. 64–19-7) (100% purity), phosphoric acid (CAS No.) (% purity), potassium hydrogen phthalate (CAS No. 877–24-7) (≥ 99.5% purity), and sodium hydroxide (CAS No. 1310–73-2) (≥ 97.0% purity) were obtained from Merck. Illovo provided ethanol (CAS No. 64–17-5) (99.9% purity). Silver nitrate, sodium chloride, sodium bromide, potassium iodide, and triethanolamine were supplied by Glass World, South Africa. All chemicals were used as received.

### Methods

#### Synthesis of photocatalysts

The Ag/AgCl catalyst was synthesized using a hydrothermal method adapted and modified from Kuai et al. (2010). In summary, 105 mg of AgNO_3_ was dissolved in 30 mL of deionized water, thereafter 0.0016 mol of NaCl was added to the solution. The solution was vigorously stirred for 10 min, before placing it in an autoclave set at 120 °C for 2 h. The resulting product was cooled to room temperature and washed several times with deionized water and ethanol. Ag/AgCl was obtained by dispersing the obtained product in 10 mL of deionized water and irradiating it under visible light for 3 h to convert Ag^+^ ions on the surface region of AgCl to Ag^0^ species. The final catalyst was obtained by drying the collected material and drying it for 12 h at 60 °C. Ag/AgBr and Ag/AgI catalysts were prepared using the same procedure, however, NaBr and KI were used as halogen sources, respectively.

#### Characterization

Phase structural patterns, crystallinities, and crystallite sizes of the Ag/AgX (X = Cl, Br, I) were evaluated by XRD using a PANalytical X’Pert Pro powder diffractometer, equipped with Co-Kα radiation source (λ = 1.789 Å). The data was collected over the 2θ range of 5° − 90°. The mineralogy was investigated through the selection of the best-fit pattern from the Inorganic Crystal Structure Database (ICSD) to the obtained diffraction pattern, through the X’Pert Highscore plus software.

An XPS spectrometer (Thermo ESCAlab 250Xi) was used to determine the surface composition of Ag/AgX (X = Cl, Br, I) as well as establishing whether elemental Ag was indeed formed on the surface of the AgX samples. The photoelectrons were excited by the monochromatic Al-kα (1486.7 eV) radiation as the excitation source and further detected with a hemispherical analyser. The analyser was operated with an energy of 100 eV for the survey spectra and the accumulation spectra of the core levels operated at 20 eV.

The specific surface area and pore size distribution measurements of the photocatalysts were determined by N_2_ adsorption/desorption method developed by Brunauer, Emmett, and Teller (BET). The equipment used was a Micrometrics Tristar 3000 BET analyser. The samples were degassed at 150 °C for 24 h, for the removal of moisture or any other extraneous materials present.

The morphology of the samples was investigated using Scanning Electron Microscopy (SEM). The images were captured using a Zeiss Ultra Plus FEG scanning electron microscope, equipped with the Oxford instruments detector and Aztec 3.0 Software SP1. The elemental analysis was performed using SEM–EDS. The samples were coated with carbon prior to analysis and the micrographs were performed at 3 kV.

The optical properties of the as-prepared samples were investigated through Ultraviolet–visible spectroscopy using the UV-1600PC spectrophotometer with a grating 1200 line/mm silicon photodiode detector and a tungsten light source. Samples were analysed at room temperature at a range of 200 − 800 nm. The photoluminescence (PL) excitation and emission were measured on the Horiba Scientific Fluoro Max 4 Spectrofluorometer. The measurements were conducted using a 150-W CW ozone-free xenon arc lamp as an excitation source. Samples were excited at 360 nm and measured within the wavelength range of 3000 − 800 nm.

#### Photocatalytic tests

The degradation efficiency of the as-synthesized Ag/AgX (X = Cl, Br and I) catalysts was evaluated using simulated 2,4-dichlorophenol (2,4-DCP) contaminated wastewater under UV and visible light irradiation. The reactor set up comprised of 400 mL glass beaker placed on a magnetic stirrer, under a light source. A 72 W LED lamp with a wavelength ranging from 380–800 nm was used as the visible light source, while a 36 W LED (PHILIPS TUV 36 W/C36 T8) lamp served as the UV light source. 250 mL of 10 mg/L 2,4-DCP solution was added to the beaker, thereafter, 125 mg of photocatalyst was added and the resulting suspension was stirred in the dark for 1 h to reach the adsorption–desorption equilibrium. The photocatalytic degradation was allowed to take for 5 h, during this period, 2 mL aliquots were sampled at predetermined time intervals. These were subsequently analysed after centrifugation and filtration to remove the solid catalyst particles.

#### Analytical methods

The concentration of 2,4-DCP was determined using a Waters 2695 High Pressure Liquid Chromatography (HPLC) instrument fitted with a 2489 UV/Visible detector. A Waters PAH C18 (250 × 4.6 mm) column operated at 25 °C was used to attain separation of the various components. The mobile phase was 60:40 (v/v) acetonitrile and deionized water and the flow rate at 1 mL min^−1^. The extent of 2,4-DCP mineralization was evaluated by measuring the total organic carbon (TOC) using a Shimadzu TOC-V Analyser.

## Results and discussion

### X-ray diffraction (XRD) of the catalyst

**Fig. 1:**
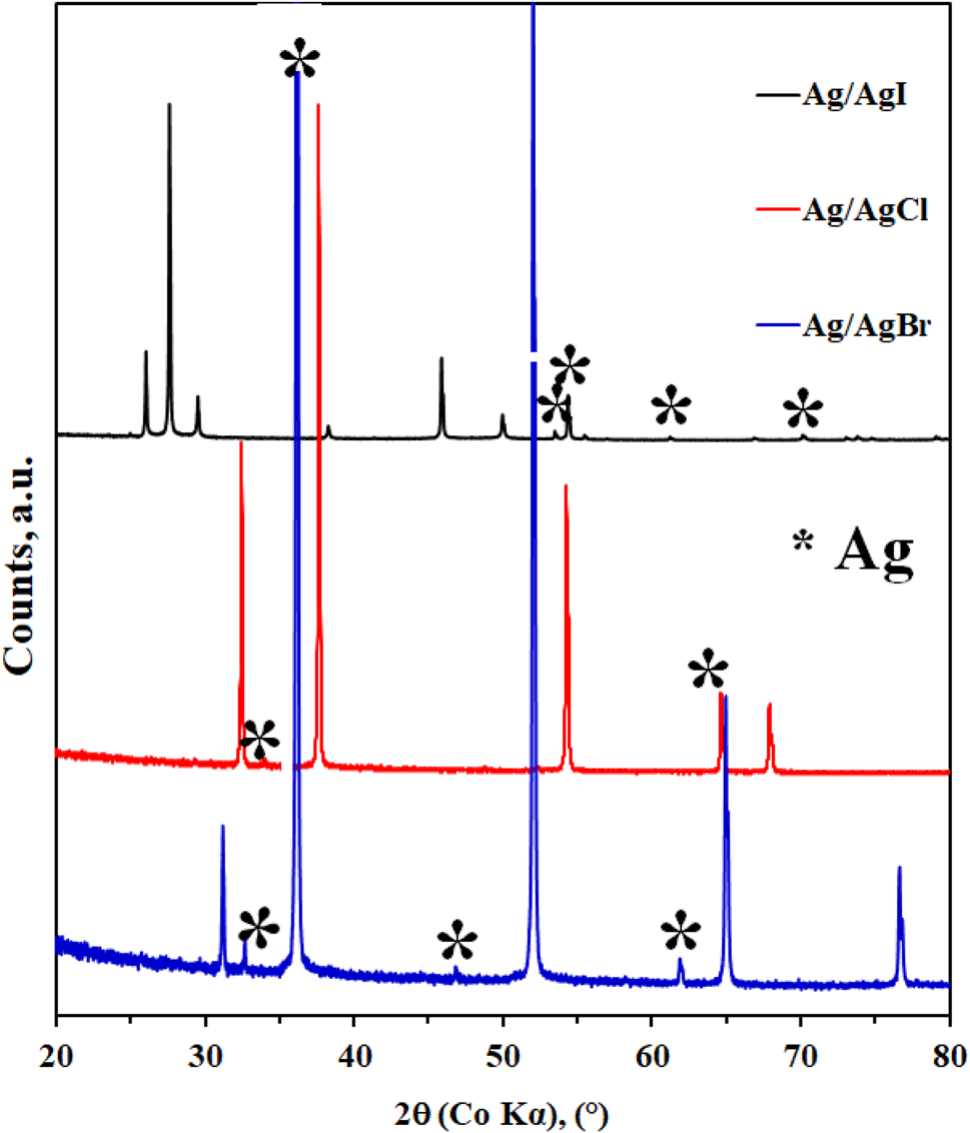
X-ray diffraction patterns of synthesized **a** Ag/AgCl, **b** Ag/AgBr and **c** Ag/AgI

Figure 1 shows the X-ray diffractograms of the Ag/AgX (X = Cl, Br, I) photocatalysts. The presented diffraction peaks of the samples were all sharp and intense, indicating the high degree of crystallinity for all the synthesized material. The XRD pattern of Ag/AgCl displayed cubic phase of AgCl, with distinct diffraction peaks at 2θ of 32.84°, 38.16°, 54.78° and 67.78° which can be assigned to (200), (220), (311) and (400) planes (JCPDS card No. 31–1238) (Wang et al. 2012), and the peaks at 34.36°and 64.97° are attributed to the diffraction of the (111) and (220) planes of metallic Ag (JCPDS card No. 04–0783) (Corsino and Balela 2017). The XRD patterns of Ag/AgBr showed peaks at 2θ values of 30.95°, 52.85°, 65.75° and 78.72° which can be associated with (200), (220), (400), (420) reflections of the cubic AgBr (JCPDS card No. 79–0148), and the peaks at 33.215°, 37.52°, 46.98° and 62.76° can be assigned to (122), (111), (200) and (220) of Ag (JCPDS card No. 65–2871) (Dai et al. 2013). The XRD patterns of Ag/AgI demonstrated the peaks at 2θ values of 24.15°, 26.85°, 29.46°, 36.51°, 46.54° which were associated with planes (100), (002), (101), (110) and (112) (JCPDS card No. 09–0374) (Chen et al. 2015) and it was observed peaks at 2θ values of 50.05°, 54.01°, 54.48°, 61.76°, and 70.61° which can be assigned to (231), (142), (241), (220) and (311) of metallic Ag (JCPDS card No. 04–0783) (Cheng et al. 2012; Meng 2015). In conclusion, the characteristic peaks of silver halide (AgX; X = Cl, Br and I) and metallic silver peaks were detected in all the samples.

### Chemical state and composition

**Fig. 2:**
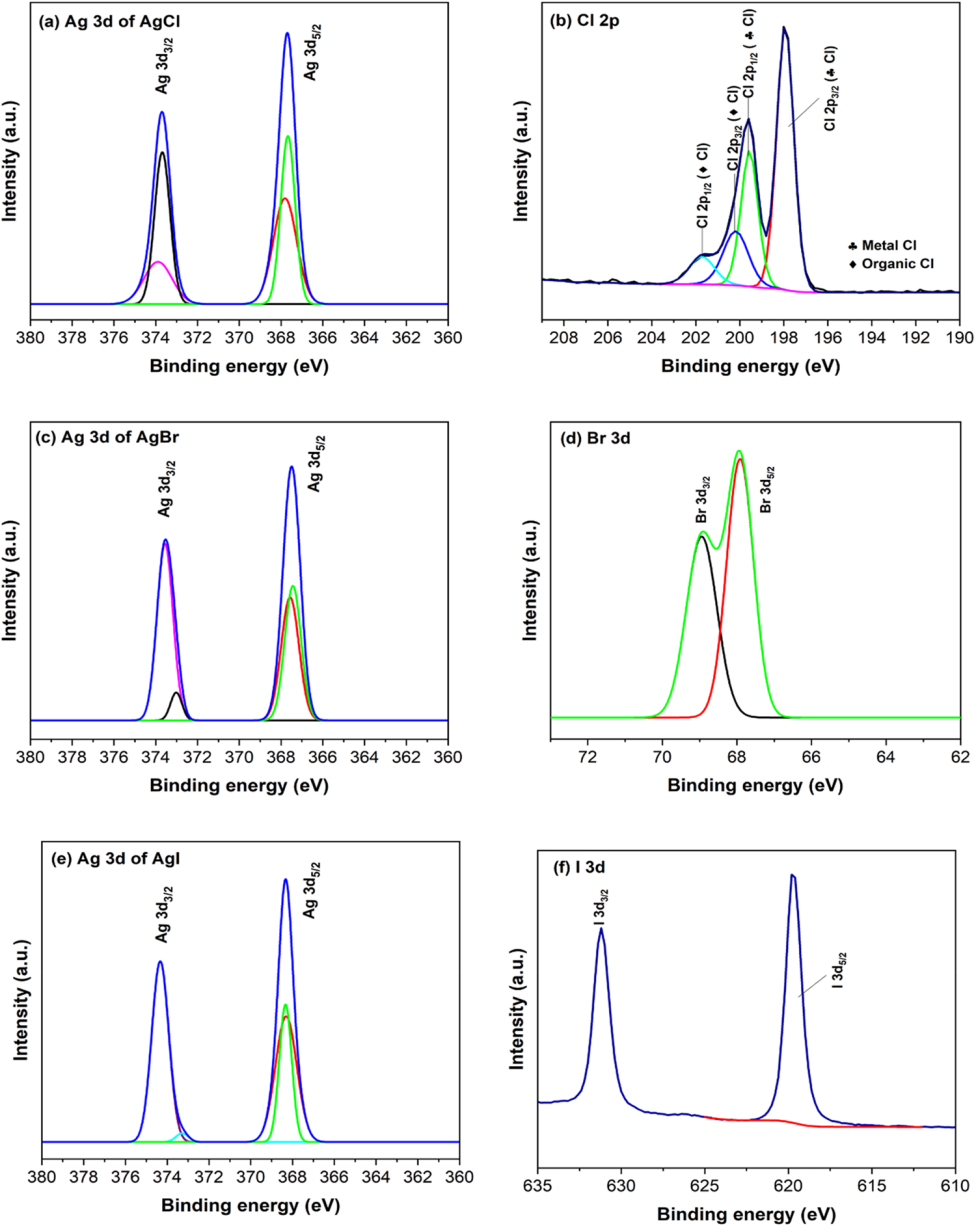
Overview XPS spectrum of the as-prepared Ag/AgCl, Ag/AgBr and Ag/AgI nanoparticles. The XPS narrow-scan spectra represents (**a**) Ag 3d and (**b**) Cl 2p of Ag/AgCl; (**c**) Ag 3d and (**d**) Br 3d of Ag/AgBr; (**e**) Ag 3d and (**f**) I 3d of Ag/AgI

The surface chemistry of the various catalysts was evaluated using XPS analysis. The main purpose for conducting this analysis was to confirm the presence of elemental silver in the material. Figure 2a, c and e, showed that all the Ag/AgX samples contained two typical 3d peaks located at ~ 367 and ~ 374 eV, which are ascribed to Ag 3d_5/2_ and Ag 3d_3/2_ binding energies (Mao et al. 2018; Liang et al. 2015; Liu et al. 2017; Ai et al. 2013). The Ag 3d_5/2_ and Ag 3d_3/2_ peaks can be further deconvoluted into two peaks. In Fig. 2a, the peaks at 367.7 and 373.7 eV were attributed to Ag^+^ ions from the AgCl crystal while those at 367.8 and 373.9 eV were attributed to metallic Ag^0^. Similar peak assignments were noted in Fig. 2c and e, for the AgBr and AgI, respectively. The high-resolution XPS spectra of Cl 2p, Br 3d and I 3d are presented in Fig. 2b, d and f, respectively. In Fig. 2b, the peaks at 198.0 and 199.6 eV were attributed to Cl 2p1/2 and Cl 2p3/2 of non-metal Cl, whereas the peaks at 200.2 (Cl 2p3/2) and 201.7 eV (Cl 2p1/2) were attributed to Cl. It should be noted that the XPS results presented in Fig. 2b indicate the presence of organic chlorine even though the primary Cl-bearing precursor material was sodium chloride. The appearance of organic Cl was postulated to have resulted from possible contamination in the reagents. The Br 3d spectrum for AgBr (Fig. 2d), presented binding energies of 67.9 and 69.0 eV, for Br 3d_5/2_ and Br 3d_3/2_, respectively. Figure 2f presents the 3d spectra binding energy attributed to I 3d_5/2_ and I 3d_3/2_ of AgI, as 619.8 and 631.3 eV are, respectively.

### Textural characterization via N_2_ adsorption/desorption at 77 K

**Fig. 3:**
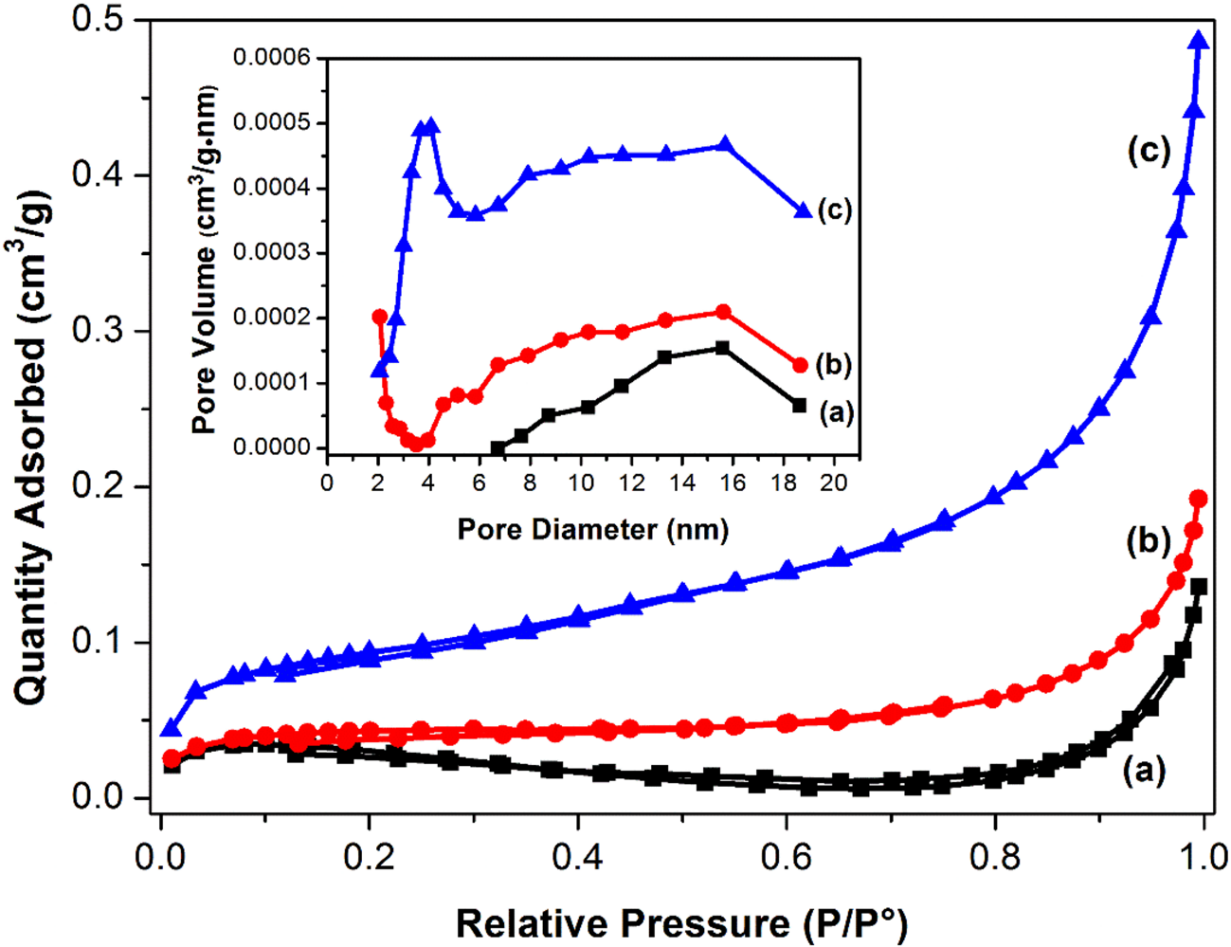
N_2_ adsorption/desorption isotherms and inset corresponding pore size distribution curves of the as-prepared photocatalysts: **a** Ag/AgCl, **b** Ag/AgBr and **c** Ag/AgI

Figure 3 illustrates the adsorption/desorption isotherms of the photocatalysts Ag/AgCl, Ag/AgBr and Ag/AgI, respectively. The samples presented the same type IV isotherm, typical of mesoporous materials, in which the principal characteristic is a hysteresis loop, and the lack of limitation of nitrogen adsorption at high values of P/P_0_ (Sing 1985; Mapossa et al. 2020). The samples had a type 3 hysteresis loop (H3), which is categorized by different evaporation and condensation paths between the adsorption and desorption processes (Sing 1985; Korichi et al. 2012) and constitute a pore formation with wedge, parallel plate forms. These characteristics suggest that the samples may display multilayer formations and the presence of interparticle mesopores, which originate from the agglomeration of small crystallites (Korichi et al. 2012). The pore size distributions of Ag/AgCl, Ag/AgBr and Ag/AgI were relatively narrow with average pore sizes of 12.4, 6.8 and 5.8 nm, respectively. The BET surface area of the Ag/AgCl, Ag/AgBr and Ag/AgI corresponded to 0.09, 0.14 and 0.33 m^2^/g, respectively. These values were surprisingly lower than those reported in literature for similar materials. For example, Lin et al. (2012) reported BET surface areas of 17.307 and 8.248 m^2^/g for Ag/AgBr and Ag/AgI respectively. While Zai et al. (2017) reported a surface area of 1.40 m^2^/g of AgCl, which is 15.5 times higher than the obtained surface area. It is however worth noting that different synthesis methods were used in these studies, this possibly affected both the particle size and morphology of these materials which in turn influences the BET surface area.

### Scanning electron microscopy (SEM)/energy dispersive spectroscopy (EDS)

**Fig. 4:**
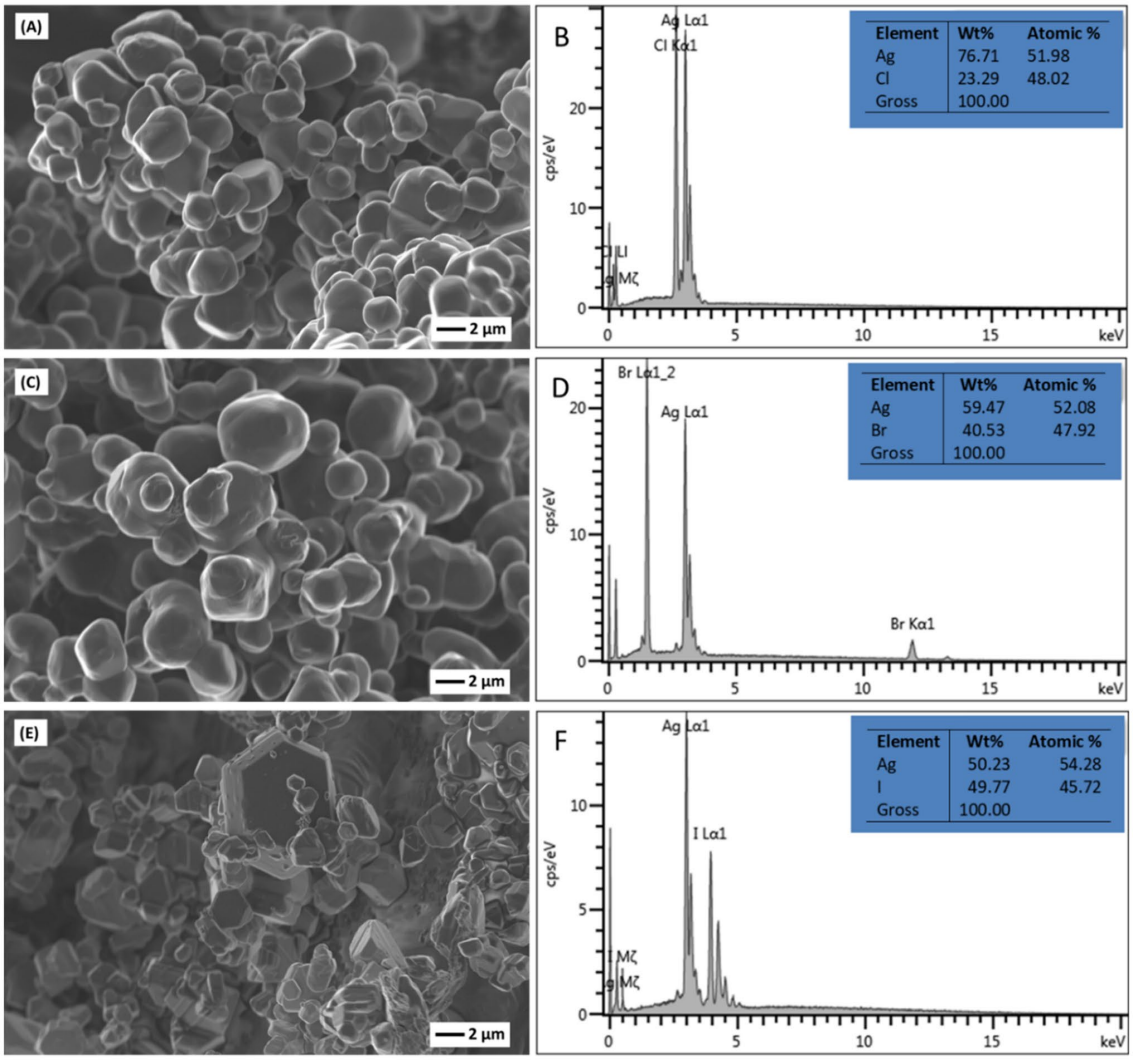
SEM images of **a** Ag/AgCl, **c** Ag/AgBr and **e** Ag/AgI as well as EDS spectrum of **b** Ag/AgCl, **d** Ag/AgBr and **f** Ag/AgI

The SEM micrographs of the as-synthesized Ag/AgX (X = Cl, Br, I) are presented in Fig. 4a, c and e. Both Ag/AgCl and Ag/AgBr were characterized by irregular and near-spherical particles, with an average primary particle size of 2 µm, which formed agglomerates (Fig. 4a and b). The morphology of the Ag/AgI featured stacked polygonal plates which were also in the micron range (Fig. 4e). The formation of agglomerates with a non-uniform distribution of particles observed in this study is a characteristic of surfactant-free precipitation reactions in aqueous media (Reddy et al. 2015). The size and distribution of Ag particles are difficult to differentiate because AgX is quickly decomposed under the high-energy beam of electrons.

Figure 4b, d and f show the elemental composition of the synthesized Ag/AgCl, Ag/AgBr and Ag/AgI catalysts obtained from SEM/EDS measurements. The absence of additional elements other than the desired ones confirmed the purity of the materials. Additionally, the silver to halide atomic % ratio for all the materials was approximately equal to the theoretically expected values.

### Optical properties

**Fig. 5:**
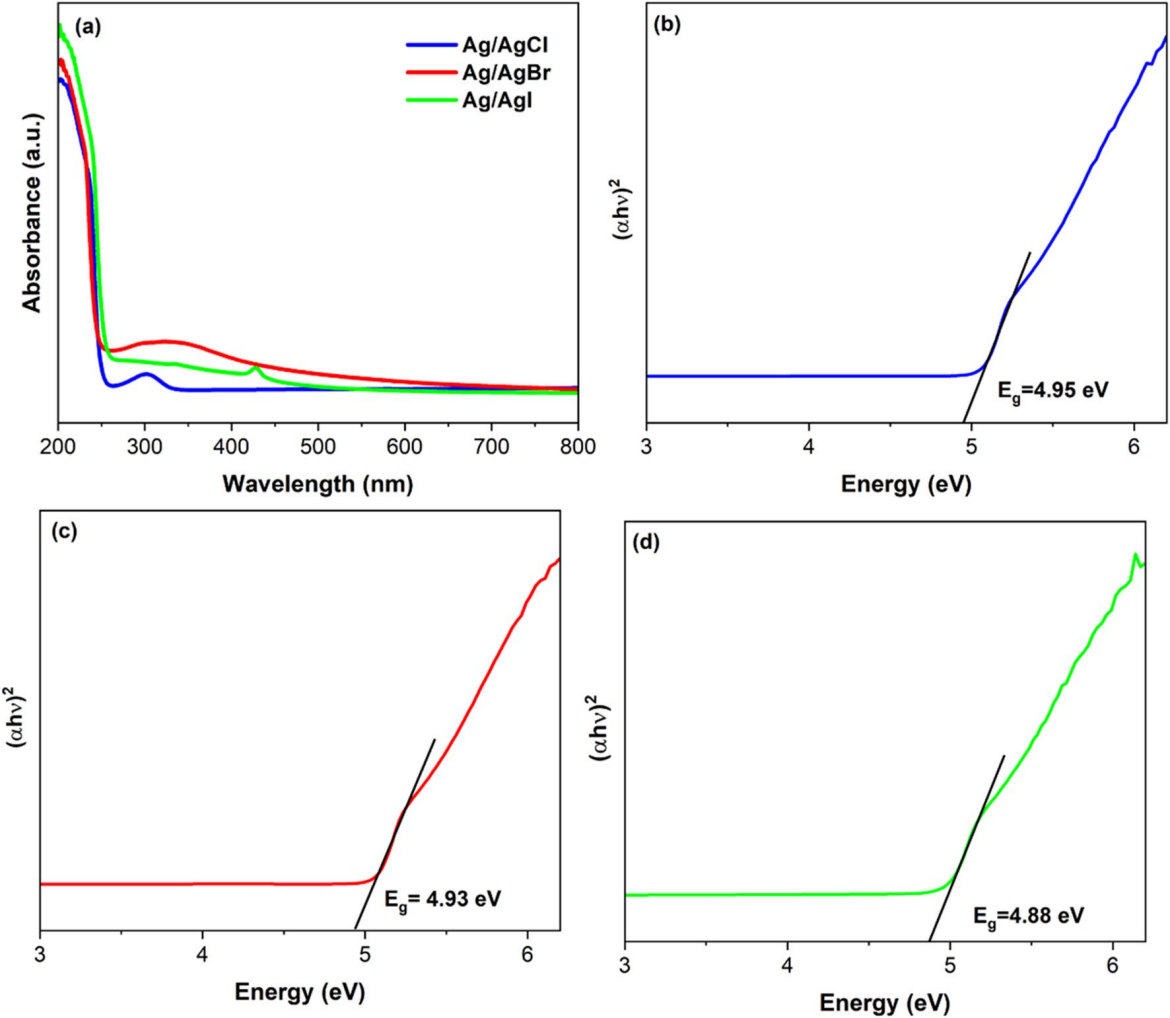
UV–visible diffuse reflectance spectra of the **a** photocatalysts and band gap energy of **b** Ag/AgCl, **c** Ag/AgBr and **d** Ag/AgI

The AgX (X = Cl, Br, I) samples exhibited distinct adsorption in the UV region but limited within the visible light region (Fig. 5a). The AgCl sample presented a peak at 305 nm which corresponds to indirect exciton transition (An et al. 2010). The adsorption edge of AgBr was estimated to be at ca. 258 nm with an extended wavelength from 265 to 429 nm. AgI presented a sharp absorption peak with maximum absorption occurring around 428 nm, this was attributed to the characteristic band of the material induced by the forbidden transition (4d^10^ to 4d^9^5s^1^) permitted by the tetrahedral symmetry of the Ag^+^ ion site (Reddy et al. 2015). The limited absorption in the visible light region suggests that the irradiated AgX (X = Cl, Br, I) catalysts produced trace amounts of metallic Ag. The band-gap energies of the Ag/AgX nanoparticles were calculated by plotting the relation between the square of the Tauc function (αhν)^1/2^ and energy in electron volts as shown in Fig. 5b, c and d. The band-gap energies of AgCl, AgBr and AgI were estimated to be 4.95, 4.93 and 4.88 eV, respectively. These were markedly different from those reported by Victora (1997) who found band gap energies of 5.6 eV, 4.3 eV and 2.8 eV for, AgCl, AgBr and β-AgI, respectively. The variation could be attributed to a number of factors such as the crystal structure of the material, morphology, defect states and charged impurities of the catalyst. In additional, the bang gap energy is also affected by temperature of preparation of the photocatalysts. The band gap of a semiconductor decreases with an increase in temperature. The spacing between atoms increases when the amplitude of the atomic vibrations increases owing to the increased thermal energy. The increased interatomic spacing decreases the potential seen by the electrons in the semiconductor, which in turn reduces the size of the energy band gap (Chauhan et al. 2015).

### Photoelectrochemical properties

**Fig. 6:**
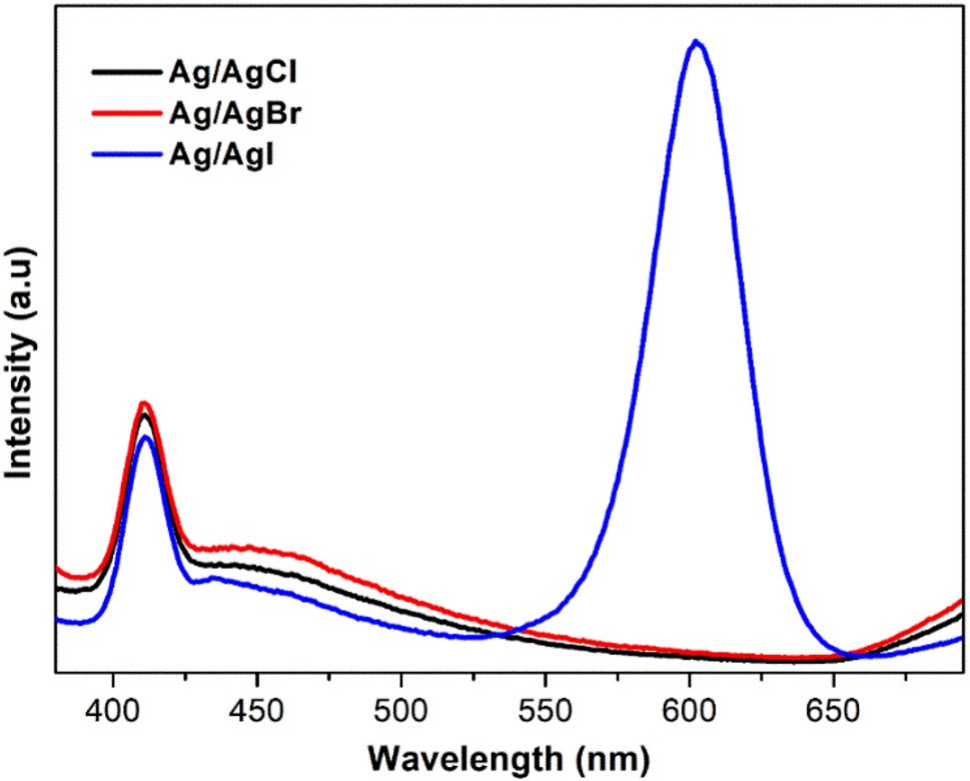
Photoluminescence spectra of the as-prepared Ag/AgCl, Ag/AgBr and Ag/AgI photocatalysts

Figure 6 shows the photoluminescence of the Ag/AgX (X = Cl, Br, I) nanoparticles at an excitation wavelength of 360 nm. The spectra of Ag/AgCl and Ag/AgBr exhibited an emission peak centred at 411 nm which was ascribed to the recombination of electron–hole pairs (Han et al. 2014; Wang 2016). Moreover, Ag/AgI presented two peaks at around 411 and 602 nm which may be attributed to distant pair donor–acceptor (D-A) recombination mediated by the density of deep trap states involving exciton-phonon interactions or crystalline defects or impurities (Reddy et al. 2015). The high photoluminescence intensity of Ag/AgI at 602 nm demonstrated high recombination efficiency of the photogenerated electron–hole which can negatively affect the photocatalytic activity of Ag/AgI. As summary, the decreased PL intensity indicated that the catalysts possess a longer carrier lifetime because of the improvement in the photogenerated electron–hole pair separation, resulting in reduced recombination rate, which is advantageous for the increased photocatalytic activity.

### Photocatalytic studies

**Fig. 7:**
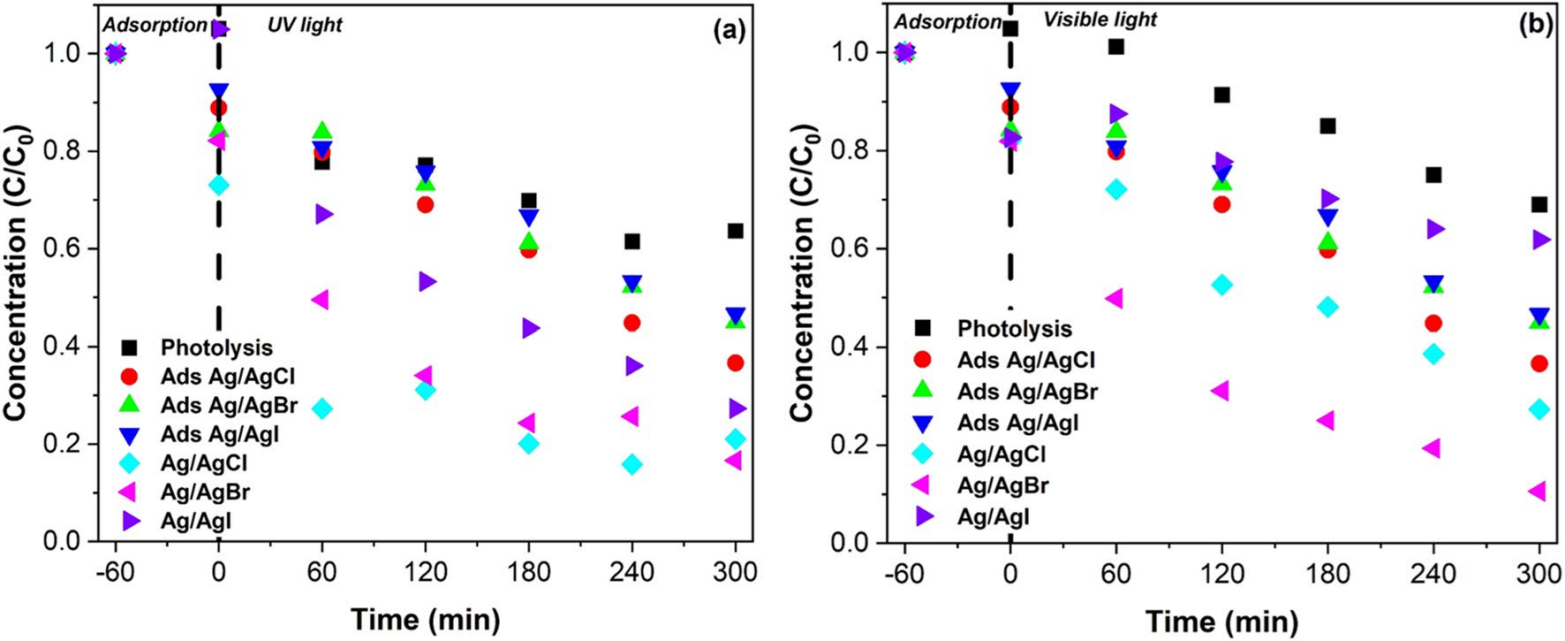
Photocatalytic degradation of 2,4-DCP with as-prepared photocatalysts Ag/AgX (X = Cl, Br, I) under **a** UV-light and b visible light irradiation

Figure 7 shows the temporal concentration changes of 2,4-DCP with different as-synthesized photocatalysts. The degradation of the pollutant in the photolysis control experiments resulted in 36.4% and 31.0% removal under UV and visible-light irradiation after 300 min, respectively. Under these conditions, the degradation mechanism was postulated to occur as a result of the direct dichlorination of 2,4-DCP through a nucleophilic displacement of chlorine Kuo (1999). An additional control experiment investigated the adsorption potential of the nanoparticles. Ag/AgCl photocatalyst exhibited an adsorption of 63.4% after 300 min, which was slightly higher than Ag/AgBr (53.4%) and Ag/AgI (55.1%). The higher adsorption capacity can be attributed to the strong electrostatic attraction between the negatively charged functional groups in silver halide catalysts and the positively charged functional groups in 2,4-DPC molecules. Figure 7 also presents the photocatalytic degradation efficiencies of 2,4-DCP using the silver halides nanoparticles under UV and visible-light irradiation. Ag/AgBr photocatalyst exhibited the highest photodegradation efficiency under both UV and visible-light irradiation for 2,4-DCP removal after 300 min, with a degradation of 83.4% and 89.4%, respectively. While Ag/AgI had the lowest efficiency under both lights with approximately 72.7% removal under UV irradiation and 38.2% under visible light after 300 min of irradiation. The reduced activity for the Ag/AgI agreed with its PL results which showed a high likelihood of electron–hole recombination. Ag/AgCl presented intermediate results compared to the other materials with degradation efficiencies of 79.0% and 72.7% under UV and visible light, respectively. A study by Tian (2012) poised that the visible light response of Ag/AgCl photocatalysts was primarily due to the plasmonic adsorption of light by Ag, whereas for Ag/AgBr and Ag/AgI both Ag and AgX respond to the visible-light irradiation producing more electrons and holes. Thus, Ag/AgBr usually presents higher photocatalytic activity than Ag/AgCl. This argument correlates with the results of this study wherein the Ag/AgBr photocatalyst exhibited the highest degradation under visible light irradiation. Therefore, for this study, Ag/AgBr was chosen as the most suitable photocatalyst for degrading 2,4-DCP under visible irradiation and all further parameter optimization studies were conducted using this catalyst.

### Optimum operating conditions

#### Catalyst loading

**Fig. 8:**
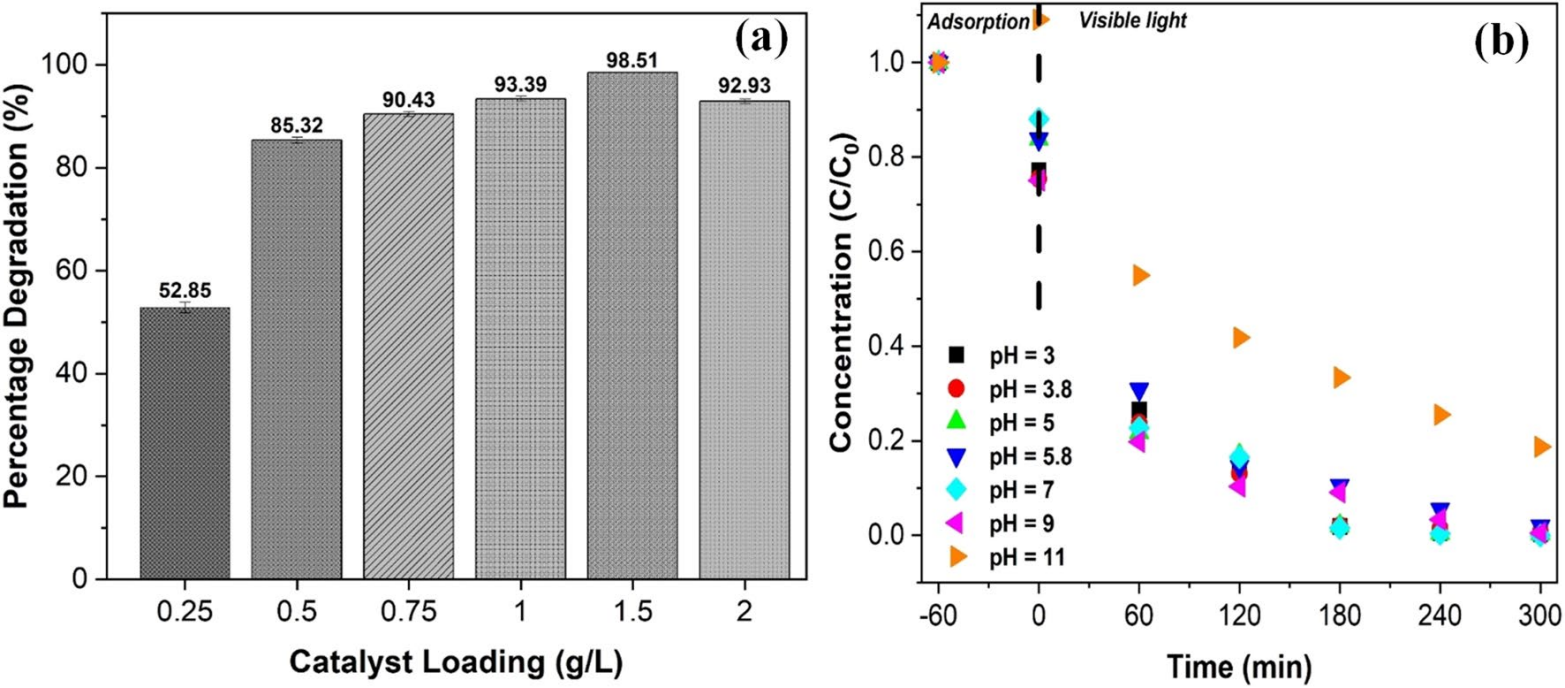
**a** Effect of catalyst loading on the degradation of 2,4-DCP using Ag/AgBr under the following conditions: 2,4-DCP concentration; 10 mg/L; visible light illumination time: 300 min, pH 5.8. **b** Effect of initial pH concentration on the photocatalytic degradation of 2,4-DCP in water using Ag/AgBr: 1.5 g/L; 2,4-DCP; 10 mg/L; illumination time: 300 min

In order to determine the optimal amount of Ag/AgBr photocatalyst required for maximum degradation, the catalyst loading was investigated in the range of 0.25 to 2 g/L at an initial 2,4-DCP concentration of and a pH of 5.8 (natural pH of 2,4-DCP). The results are depicted in Fig. 8a, the optimum Ag/AgBr loading was 1.5 g/L. Photodegradation activity increased with increasing catalyst loading from 0.25 to 1.5 g/L, this was attributed to an increase in available adsorption and photocatalytic sites (Gaya et al. 2010; Pinho and Mosquera 2013; Guillard et al. 2005). Beyond 1.5 g/L catalyst loading, the photodegradation efficiency decreased. The excess of loading of catalyst beyond the optimum may have resulted in the agglomeration of catalyst particles and generated turbidity, which resulted to the reduction in the number of active surface sites of the catalysts consequently culminating in the decrease of the photocatalytic degradation efficiency. These results followed a trend similar to literature reported by authors (Yu et al. 2000; Pinho and Mosquera 2013; Pei and Leung 2013).

#### pH effect

Figure 8b shows the effect of initial pH value on the degradation of 2,4-DCP under the following conditions: catalyst loading of 1.5 g/L, and a 2,4-DCP concentration of 10 mg/L. High degradation efficiencies were observed in the pH range of 3 to 9 (98%), with the highest degradation occurring at an initial pH of 7. Therefore, it suggests that the presence of abundant H^+^ in the system was conducive to the catalytic reaction via promoting the turnover frequency (TOF) (Peng et al. 2023). When the initial pH value increased to 11, degradation decreased marginally to 81.3%. These results indicated that Ag/AgBr may be applied over a wide pH range without affecting the efficiency of the catalyst. Acidic and weakly acidic conditions have been reported to be advantageous in removing multiple contaminants, as they promote the generation of free radicals and oxidation potential of the hydroxyl radical (Lai et al. 2021). Additionally, 2,4-DCP remains in its molecular state (pKa of 7.89) at lower pH which makes it more susceptible to reacting with the free radicals (Gaya et al. 2010). Increasing the pH to the alkali region leads to an increase in hydroxide ion concentration; consequently, some hydroxyl radicals combine with the OH^-^ ions forming water and ultimately decreasing the amount of free radicals available for degradation (Zhang et al. 2018).

#### Effect of pollutant concentration

**Fig. 9:**
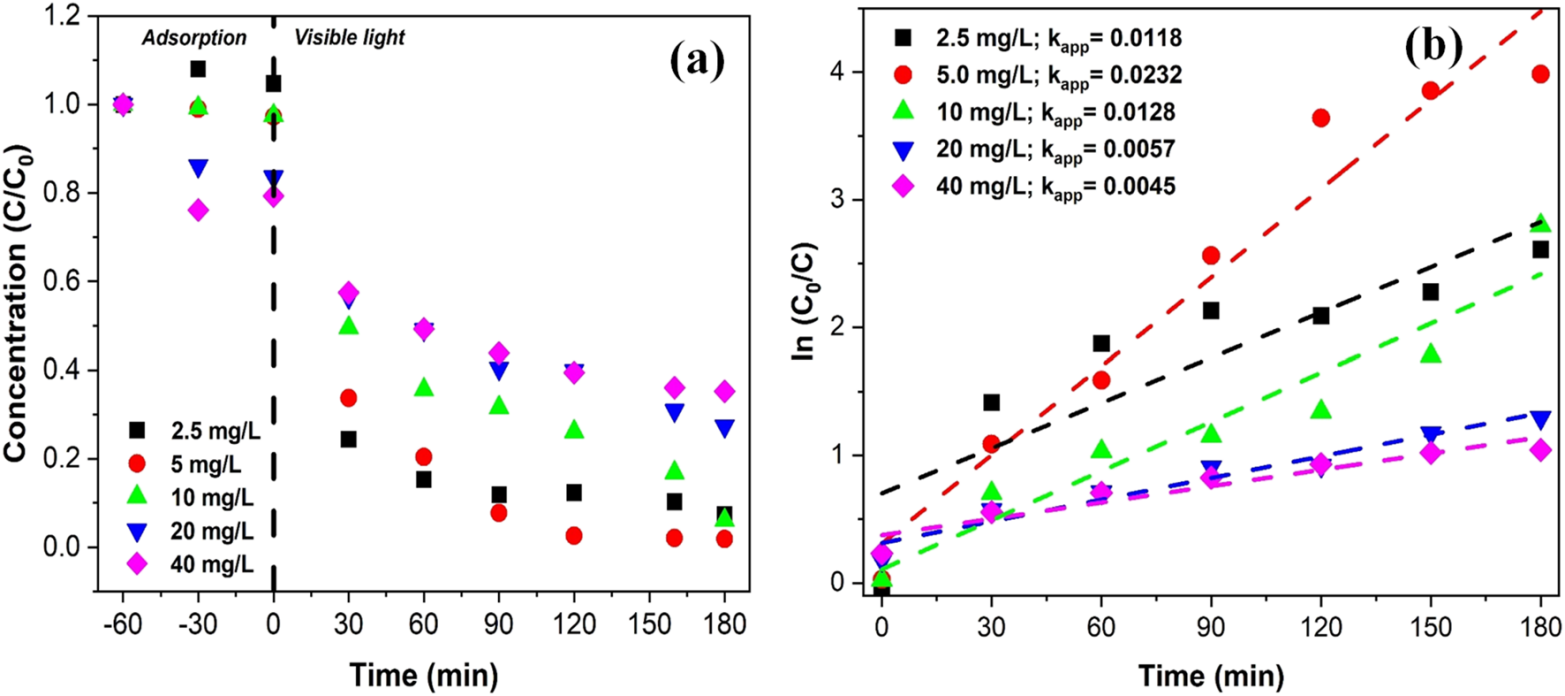
**a** Photocatalytic degradation with different initial concentration of 2,4-DCP using Ag/AgBr under visible light irradiation. **b** Pseudo-first-order reaction kinetics of 2,4-DCP at various initial 2,4-DCP concentrations; catalyst loading 1.5 g/L; pH 5

Figure 9 shows the influence of concentration on the reaction kinetics of 2,4-DCP degradation. Usually, various studies have demonstrated that the increased concentration of the pollutant increases the removal rates. This is because, with increasing pollutant concentration, the probability of collision between pollutants and the surface of the photocatalyst will be higher. Consequently, this increases the degradation rate due to the very short lifetime of the hydroxyl radicals, which are responsible for oxidizing the organic pollutants (Nezamzadeh-Ejhieh and Khorsandi 2010; Ajoudanian and Nezamzadeh-Ejhieh 2015; Nezamzadeh-Ejhieh and Shahriari 2014). However, when the substrate concentration increases to a certain extent, more intermediates will probably be generated and adsorbed on the photocatalyst surface since the photo-degradation process is non-selective, and thus, the decomposition of the pollutants as well as the obtained intermediates will take place concurrently by the produced hydroxyls and superoxide radicals. As a result, active sites on the photocatalyst are partially compromised and hence, incurring a slower adsorption rate, which eventually leads to a drop in the overall degradation rate. In short, it can be deduced that at low pollutant concentrations, the degradation rate is less dependent on the number of catalytically active sites. Instead, it is proportional to the substrate concentration by apparent first-order kinetics. Additionally, the molecular structure of the pollutants can be one of the factors affecting their degradation extent, which has been discussed by authors (Nezamzadeh-Ejhieh and Karimi-Shamsabadi 2014; Jafari and Nezamzadeh-Ejhieh 2017; Babaahamdi-Milani and Nezamzadeh-Ejhieh 2016; Roushenas et al. 2018).

Therefore, in this present study, degradation efficiency decreased with increasing pollutant concentration. Increasing the concentration of 2,4-DCP results in an increase in the number of 2,4-DCP molecules absorbed on the surface of the Ag/AgBr catalyst. This reduces the number of active sites available on the material. It has also been reported that increasing the concentration of 2,4-DCP in solution may cause a reduction in the photo-absorption capacity of Ag/AgBr particles, leading to reduced photodegradation efficiency (Liang et al. 2019).

Besides that, it can be explained that when the pollutant concentration increases, the pollutant around the photocatalyst creates a protective effect for another pollutant that prevents their destruction. In addition, an increase in the concentration of pollutants around the catalyst causes absorption and scattering of light, which, as a result, reduces the degradation efficiency of the catalyst and increases the competition for adsorption on the catalyst, which causes saturation of the catalyst. This effect is corroborated by authors (Gaya and Abdullah 2008; Hussain et al. 2011; Hadi et al. 2023).

The intrinsic kinetics of the degradation reaction were modelled using the Langmuir–Hinshelwood model (Eq. (1)) (Fan et al. 2018; Kumar et al. 2008; Melián et al. 2007):

1 $$\mathit{ln}\left(\frac{C}{{C}_{0}}\right)=-{k}_{app}t$$

where C_0_ represents the initial concentration of water pollutants (mg L^−1^), and k_app_ is the apparent rate constant (min^−1^).

The model was a good fit for most of the data with *R*^2^ values ranging from 0.95 to 0.97. The rate constants followed a trend similar to the one observed for degradation as the values decreased with increasing 2,4-DCP concentration, 0.0118 min^−1^ for the 2.5 mg/L solution and 0.0045 min^−1^ for the 40 mg/L solution.

**Fig. 10:**
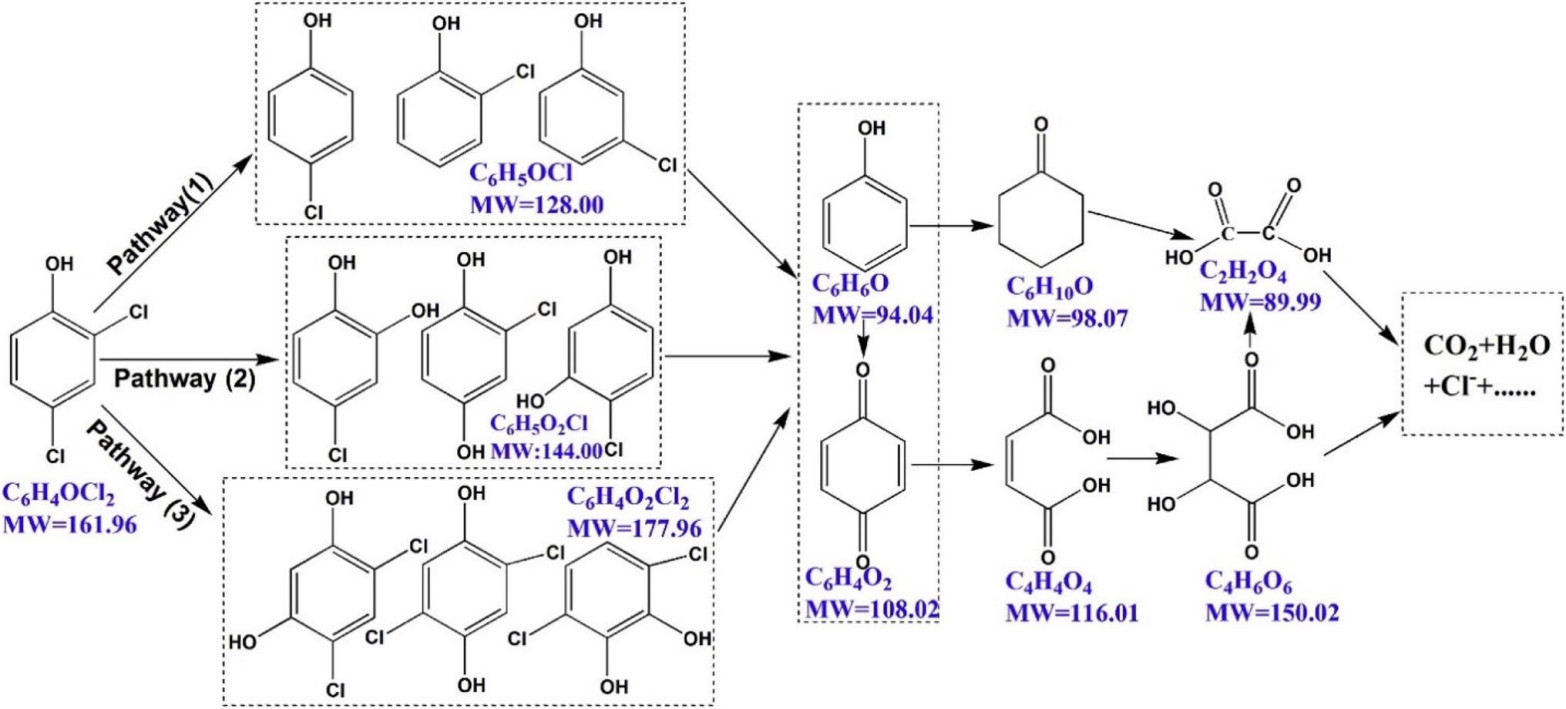
Scheme illustrating the proposed mechanism of 2,4-DCP degradation pathway on Ag/AgX photocatalysts

It is worth noting that a total organic carbon (TOC) analysis was conducted to evaluate the extent of mineralization of 2,4-DCP degraded under optimized conditions of 2,4-DCP concentration of 10 mg/L, pH 5 and catalyst loading of 1.5 g/L under visible light irradiation. From the TOC analysis demonstrated that approximately 26.9% of 2,4-DCP was mineralized while 99.7% of degradation of 2,4-DCP was achieved after 300 min. This indicated the possible formation of intermediate products during the breakdown of the primary 2,4-DCP molecule. Based on the literature (Zhang et al. 2018; Humayun et al. 2019; Chen et al. 2017), a reaction pathway for the degradation of 2,4-DCP was proposed in Fig. 10. The pathway follows three routes wherein the first pathway of dechlorination proposes that the aromatic ring was attacked by hydroxyl radicals to form p-chlorophenol and o-chlorophenol (C_6_H_5_OCl, MW 128.00) dechlorination products (Zhang et al. 2018). In the second pathway, the dechlorination products were consistently attacked by hydroxyl radicals in the reaction solution (Tang and Huang 1996). The hydroxyl radical was added onto the position of the dechlorination reaction, forming dihydroxychlorobenzene and its isomers (C_6_H_5_O_2_Cl, MW 144.00). In the third pathway, the aromatic hydroxylation involving the generation of hydroxyl radical in solution is exposed to plasma oxidation (Constantin et al. 2018). Due to the electron–hole behaviour of the phenolic –OH group and the electrophilic hydroxyl radical, the (C_6_H_5_O_2_Cl_2_, MW 177.96) (Deborde and Von Gunten 2008). After dechlorination and hydroxylation, the intermediates were further oxidized to form phenol (C_6_H_6_O, MW 94.04) (Tang and Huang 1995) and 1,4-benzoquinone (C_6_H_4_O_2_, MW 108.02) (Tang and Huang 1996). The resulting phenol could further be oxidized to cyclohexanone (C_6_H_10_O, MW 98.07) or 2,3-dihydroxybutanedioic acid (C_4_H_6_O_6_, MW 150.02). Once all the benzene rings are broken, the major products were determined to be organic acids such as oxalic acid, maleic acid and formic acid (Zazo et al. 2005). Continued reaction of these acids with free radicals results in mineralization to CO_2_ and H_2_O.

### Photochemical stability and reusability

**Fig. 11:**
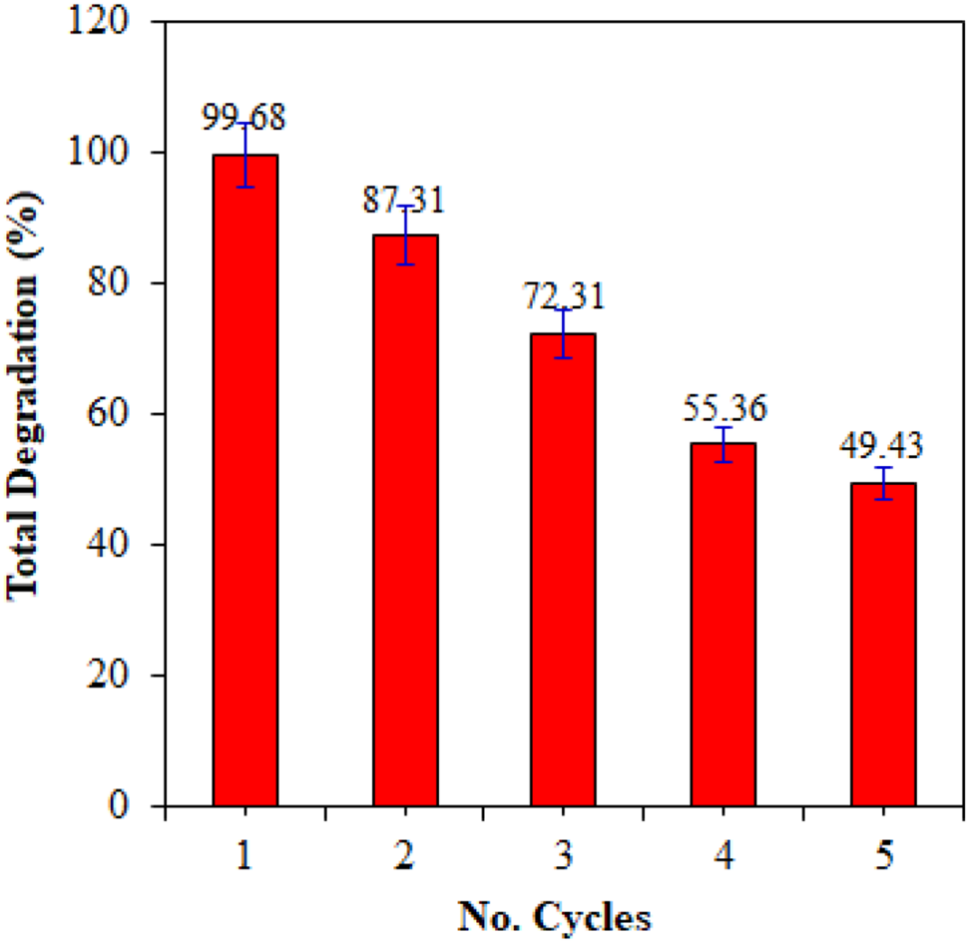
Photocatalytic reusability and stability of Ag/AgBr for the degradation of 2,4-DCP (10 mg/L); pH 5; catalyst loading: 1.5 g/L; under visible light irradiation

**Fig. 12:**
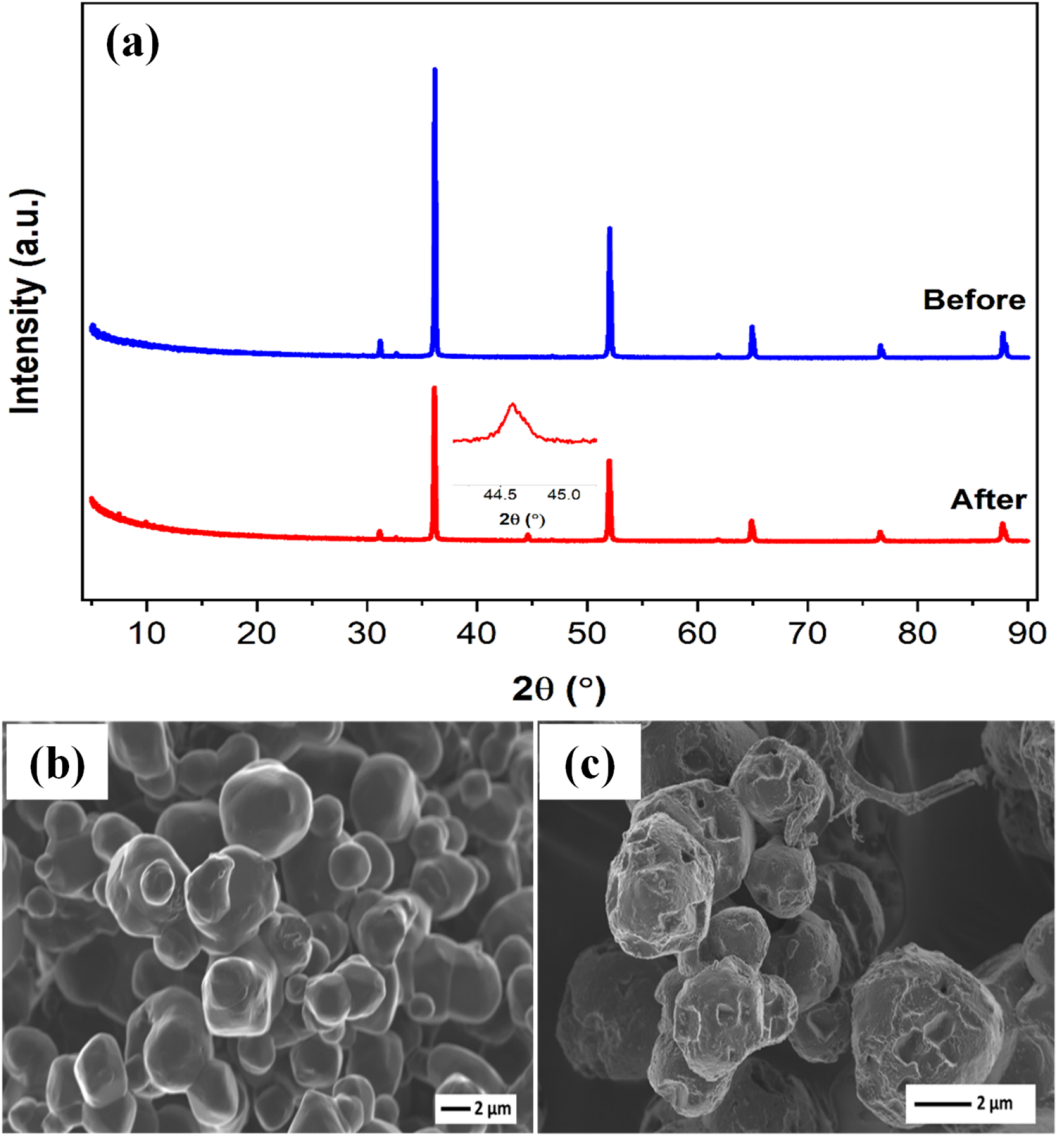
**a** XRD spectra of Ag/AgBr before and after photostability and reusability evaluation. **b** SEM images of Ag/AgBr before and **c** after photostability and reusability tests

The photocatalytic stability and reusability of the Ag/AgBr catalyst were evaluated and the results are presented in Fig. 11. Photocatalytic performance decreased by 50% after five degradation cycles. This decline may be attributed to the continuous generation of metallic Ag and accompanying photo erosion that occurs on the surface of AgBr during the photocatalytic process (Duan et al. 2021). Structural and morphological analysis conducted on the Ag/AgBr after 5 cycles confirmed this postulation. An XRD diffractogram of Ag/AgBr after 5 cycles, exhibited a distinct cubic phase Ag° diffraction peak centred at 45° which was not present in the freshly synthesized catalyst (Fig. 12a). SEM micrographs presented in Fig. 12b and c provided visual evidence of the photo corrosion that took place on the surface of the Ag/AgBr catalyst. This surface destruction reduced the number of active sites on the material resulting in decreased stability and reusability. To improve the photocatalytic reusability of these photocatalysts, a study based on the development of a core–shell structured magnetic Ag/AgX@Fe_2_O_3_ (X = Cl; Br; I) composite for 2,4-dichlorophenol (2,4-DCP) degradation is required. Therefore, from the study, the authors expect that the as-prepared Ag/AgX@Fe_2_O_3_ (X = Cl; Br; I) composite has enough magnetic properties, that after the photocatalytic reaction, it can be quickly separated from the solution by an extra magnetic field.

### Photocatalytic decomposition mechanism of 2,4-DCP

**Fig. 13:**
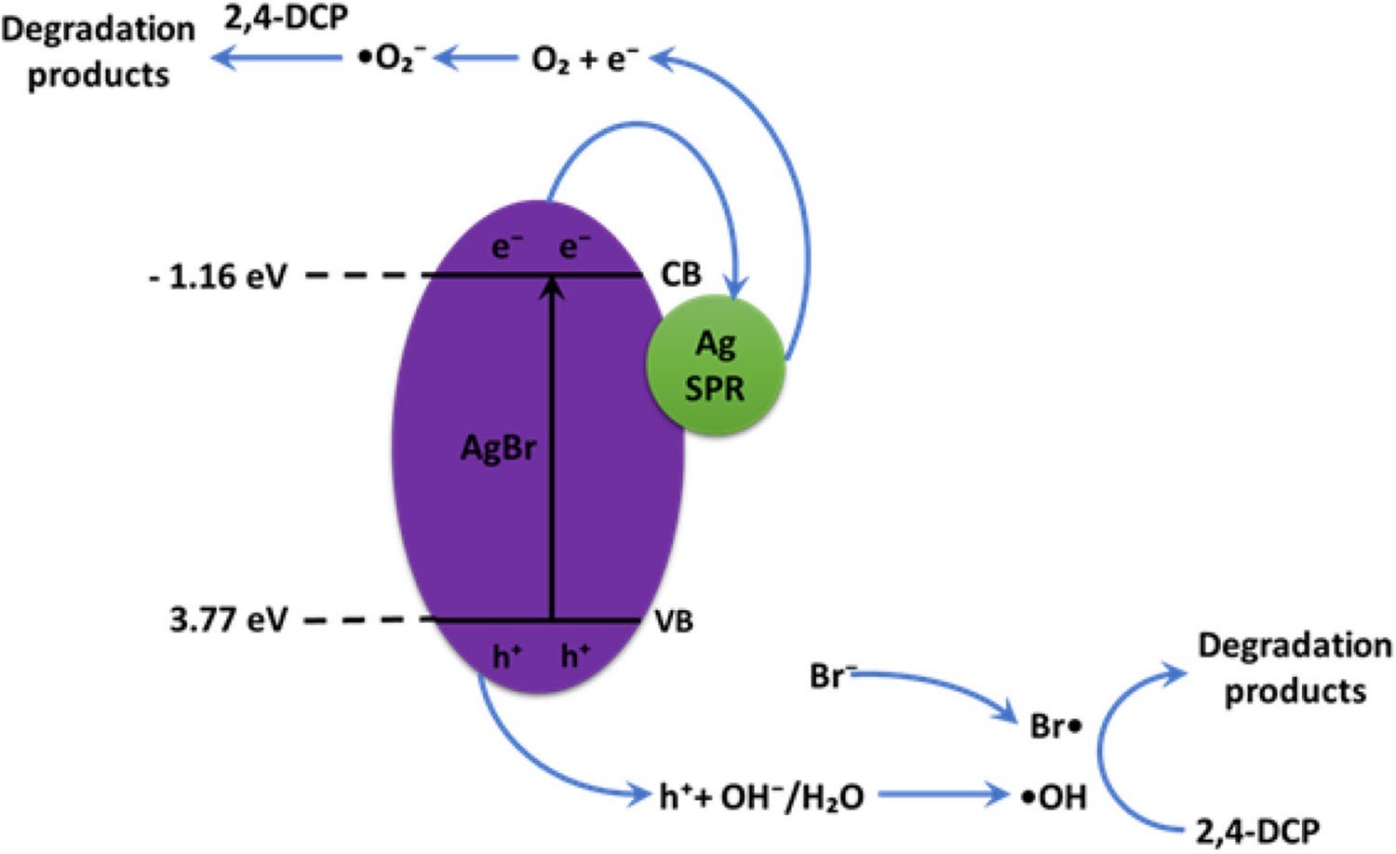
Schematic representation of the proposed mechanism for photo-generated charge transfer in Ag/AgBr under visible light irradiation

The proposed photocatalytic mechanism of Ag/AgBr in visible light degradation of 2,4-DCP is shown in Fig. 13. The photocatalytic process is governed by the oxidation potential of photogenerated holes, band gap and separation capability of photoinduced carriers in the Ag/AgBr nanoparticles as well as the Mulliken Electronegativity Relation. The valence band and conduction band potential of AgBr correspond 3.77 eV and − 1.16 eV, respectively. When Ag/AgBr nanoparticles are irradiated with visible light, AgBr generate electron–hole pairs by formulating electrons and holes in the conduction band (CB) and valence band (VB), respectively. The electron from CB band of AgBr quickly migrates to the Ag nanoparticles on the surface since the CB potential of AgBr is negative relative to the Fermi level of Ag (0.4 eV) (Tun et al. 2019).

The SPR effects of Ag nanoparticles have high reduction capability which enables them to combine with oxygen molecules leading to the formation of ^•^O_2_^-^ radicals. This effect also aids in retarding electron–hole. Moreover, the photoinduced holes in the valence band of AgBr (+ 3.77 eV) were involved in the oxidizing reaction of H_2_O/OH^-^ to generate ^•^OH radical since the potential of h^+^ in the valence band is higher than ^•^OH (+ 1.99 eV vs SHE). The formed radical is a powerful oxidizing agent capable of degrading 2,4-DCP and it intermediates directly.

AgBr is highly sensitive to light and may dissociate to form silver and bromine ions during the reaction. The photogenerated electrons reduce the Ag^+^ ions into metallic Ag, this aids in preventing further photo corrosion of the catalyst. Simultaneously, some holes may combine with Br^-^ and oxidize to Br^0^ atom, a strong oxidant capable of degrading organic pollutants (Sanni et al. 2019).

## Conclusions

In this work, the efficiency of the hydrothermal method was confirmed through different characterization strategies to determine the crystallinity, purity, morphology as well as the chemical states and composition of the synthesized Ag/AgX (X = Cl, Br, I) photocatalysts. The Ag/AgBr and Ag/AgBr presented cubic phases while the Ag/AgI presented two phases namely the hexagonal β-AgI and cubic γ-AgI. The Ag/AgCl, Ag/AgBr and Ag/AgI displayed near-spherical, irregular sphere-like and polygonal plate morphologies, respectively. The results of photocatalytic activity of photocatalysts demonstrated that all synthesized materials were activated under both UV and visible-light irradiation with Ag/AgBr exhibiting the highest overall efficiency of approximately 89.39% (under initial conditions) photodegradation under visible-light irradiation in the degrading 2,4-DCP. Under optimized catalyst loading, pH, and initial concentration of 2,4-DCP, the photocatalytic reaction efficiently removed 2,4-DCP while observing a low TOC reduction. This study demonstrated the potential use of silver halides (Ag/AgX), particularly Ag/AgBr under visible-light irradiation for the remediation of 2,4-DCP in the environment.

### Supplementary Information

Below is the link to the electronic supplementary material.

Supplementary file1 (DOCX 39 KB)

## Data Availability

All data related to this manuscript is incorporated in the manuscript.
