# Supplementary material for: Photocatalytic degradation of 2,4-dichlorophenol using nanomaterials silver halide catalysts
Source: Environ Sci Pollut Res Int. 2024 Jan 15;31(8):11857–72. doi: 10.1007/s11356-024-31921-1 (PMC10869396; doi:10.1007/s11356-024-31921-1)
Supplement: Supplementary file 1 — Supplementary file1 (DOCX 39 KB) [file 11356_2024_31921_MOESM1_ESM.docx]

**Supplementary Materials:** This data generated Figure 7. The average and standard deviation results are presented in all tables.

|  | ***Adsorption*** |  |  |  |  |  |  |  |  |  |  |  |  |  |  |  |  |  |  |
| --- | --- | --- | --- | --- | --- | --- | --- | --- | --- | --- | --- | --- | --- | --- | --- | --- | --- | --- | --- |
|  |  | Concentration | | | Normalized | | |  |  |  |  |  |  |  |  |  |  |  |  |
|  | Time | Ag/AgCl | Ag/AgBr | Ag/AgI | Ag/AgCl | Ag/AgBr | Ag/AgI |  |  |  |  |  |  |  |  |  |  |  |  |
|  | Initial Conc | 6.768 | 6.768 | 6.768 | 1 | 1 | 1 |  |  |  |  |  |  |  |  |  |  |  |  |
|  | t0 | 5.898 | 5.326 | 6.344 | 0.871454 | 0.786939 | 0.937352 |  |  |  |  |  |  |  |  |  |  |  |  |
|  | t1 | 5.204 | 5.61 | 5.225 | 0.768913 | 0.828901 | 0.772015 |  |  |  |  |  |  |  |  |  |  |  |  |
|  | t2 | 4.498 | 4.82 | 5.205 | 0.664598 | 0.712175 | 0.76906 |  |  |  |  |  |  |  |  |  |  |  |  |
|  | t3 | 3.712 | 3.817 | 4.588 | 0.548463 | 0.563978 | 0.677896 |  |  |  |  |  |  |  |  |  |  |  |  |
|  | t4 | 2.326 | 3.006 | 3.307 | 0.343676 | 0.444149 | 0.488623 |  |  |  |  |  |  |  |  |  |  |  |  |
|  | t5 | 1.936 | 2.296 | 3.095 | 0.286052 | 0.339243 | 0.457299 |  |  |  |  |  |  |  |  |  |  |  |  |
|  |  |  |  |  |  |  |  |  |  |  |  |  |  |  |  |  |  |  |  |
|  | **Adsorption** |  |  |  |  |  |  |  |  |  |  | |  | | |  |  |  |  |
|  |  | Concentration | | | Normalized | | | \|  \| \| --- \| |  |  | |  | |  |  | |  |  |  |
|  | Time | Ag/AgCl | Ag/AgBr | Ag/AgI | Ag/AgCl | Ag/AgBr | Ag/AgI |  |  |  | |  | |  |  | |  |  |  |
|  | Initial Conc | 7.001 | 7.001 | 7.001 | 1 | 1 | 1 |  |  |  | |  | |  |  | |  |  |  |
|  | t0 | 6.131 | 6.064 | 6.198 | 0.905881 | 0.895981 | 0.91578 |  |  |  | |  | |  |  | |  |  |  |
|  | t1 | 5.593 | 5.74 | 5.714 | 0.826389 | 0.848109 | 0.844267 |  |  |  | |  | |  |  | |  |  |  |
|  | t2 | 4.845 | 5.079 | 5.048 | 0.715869 | 0.750443 | 0.745863 |  |  |  | |  | |  |  | |  |  |  |
|  | t3 | 4.374 | 4.461 | 4.451 | 0.646277 | 0.659131 | 0.657654 |  |  |  | |  | |  |  | |  |  |  |
|  | t4 | 3.74 | 4.067 | 3.904 | 0.5526 | 0.600916 | 0.576832 |  |  |  | |  | |  |  | |  |  |  |
|  | t5 | 3.023 | 3.779 | 3.218 | 0.446661 | 0.558363 | 0.475473 |  |  |  | |  | |  |  | |  |  |  |
|  |  |  |  |  |  |  |  |  |  |  | |  | |  |  | |  |  |  |
|  | **Average Adsorption** | |  |  |  |  |  |  |  |  | |  | |  |  | |  |  |  |
|  |  | Average Concentration | | | Average Normalized | | | Standard deviation (SD) | | | |  | |  |  | |  |  |  |
|  | Time | Ag/AgCl | Ag/AgBr | Ag/AgI | Ag/AgCl | Ag/AgBr | Ag/AgI | Ag/AgCl | Ag/AgBr | Ag/AgI | |  | |  |  | |  |  |  |
| -1 | Initial Conc | 6.8845 | 6.8845 | 6.8845 | 1 | 1 | 1 | 0 | 0 | 0 | |  | |  |  | |  |  |  |
| 0 | t0 | 6.0145 | 5.695 | 6.271 | 0.888667 | 0.84146 | 0.926566 | 0.024343 | 0.077105 | 0.015253781 | |  | |  |  | |  |  |  |
| 1 | t1 | 5.3985 | 5.675 | 5.4695 | 0.797651 | 0.838505 | 0.808141 | 0.040642 | 0.013582 | 0.051089719 | |  | |  |  | |  |  |  |
| 2 | t2 | 4.6715 | 4.9495 | 5.1265 | 0.690233 | 0.731309 | 0.757462 | 0.036254 | 0.02706 | 0.016403039 | |  | |  |  | |  |  |  |
| 3 | t3 | 4.043 | 4.139 | 4.5195 | 0.59737 | 0.611554 | 0.667775 | 0.069164 | 0.067284 | 0.014313479 | |  | |  |  | |  |  |  |
| 4 | t4 | 3.033 | 3.5365 | 3.6055 | 0.448138 | 0.522533 | 0.532728 | 0.147732 | 0.110851 | 0.062373338 | |  | |  |  | |  |  |  |
| 5 | t5 | 2.4795 | 3.0375 | 3.1565 | 0.366356 | 0.448803 | 0.466386 | 0.113568 | 0.154941 | 0.012850788 | |  | |  |  | |  |  |  |
|  |  |  |  |  |  |  |  |  |  |  | |  | |  |  | |  |  |  |

|  |  | | | | | | | |  |  |  |  |  |  |  |  |
| --- | --- | --- | --- | --- | --- | --- | --- | --- | --- | --- | --- | --- | --- | --- | --- | --- |
|  |  |  |  |  |  |  |  |  |  |  |  |  |  |  |  |  |
|  | **UV Light Photolysis** | |  |  |  |  |  |  |  |  |  |  |  |  |  |  |
|  |  | Concentration | | |  | Normalized |  |  |  |  |  |  |  |  |  |  |
|  | Time | Ag/AgCl | Ag/AgBr | Ag/AgI | Average | Normalized | SD |  |  |  |  |  |  |  |  |  |
| -1 | Initial Conc | 6.141 | 6.141 | 6.141 | 6.141 | 1 | 1.09E-15 |  |  |  |  |  |  |  |  |  |
| 0 | t0 | 6.321 | 6.382 | 6.667 | 6.456667 | 1.051403 | 0.18469 |  |  |  |  |  |  |  |  |  |
| 1 | t1 | 5.356 | 4.511 | 4.459 | 4.775333 | 0.777615 | 0.503544 |  |  |  |  |  |  |  |  |  |
| 2 | t2 | 5.296 | 4.3 | 4.618 | 4.738 | 0.771536 | 0.508728 |  |  |  |  |  |  |  |  |  |
| 3 | t3 | 4.743 | 4.071 | 4.06 | 4.291333 | 0.6988 | 0.391193 |  |  |  |  |  |  |  |  |  |
| 4 | t4 | 4.282 | 3.645 | 3.402 | 3.776333 | 0.614938 | 0.454463 |  |  |  |  |  |  |  |  |  |
| 5 | t5 | 3.947 | 3.373 | 4.402 | 3.907333 | 0.63627 | 0.515646 |  |  |  |  |  |  |  |  |  |

|  | **Visible Light Photolysis** | |  |  |  |  |  |
| --- | --- | --- | --- | --- | --- | --- | --- |
|  |  | Concentration | | |  | Normalized |  |
|  | Time | Ag/AgCl | Ag/AgBr | Ag/AgI | Average | Normalized | SD |
| -1 | Initial Conc | 6.423 | 6.423 | 6.423 | 6.423 | 1 | 1.09E-15 |
| 0 | t0 | 6.408 | 6.758 | 7.044 | 6.736667 | 1.04883492 | 0.318536 |
| 1 | t1 | 6.239 | 6.609 | 6.659 | 6.502333 | 1.01235145 | 0.22942 |
| 2 | t2 | 6.207 | 5.314 | 6.08 | 5.867 | 0.91343609 | 0.483104 |
| 3 | t3 | 6.194 | 4.55 | 5.642 | 5.462 | 0.85038144 | 0.83665 |
| 4 | t4 | 5.155 | 4.132 | 5.164 | 4.817 | 0.74996108 | 0.593244 |
| 5 | t5 | 4.794 | 3.592 | 4.915 | 4.433667 | 0.69027972 | 0.731411 |

| **UV Light Photocatalyis** | | |  |  |  |  |
| --- | --- | --- | --- | --- | --- | --- |
|  | Concentration | | | Normalized | | |
| Time | Ag/AgCl | Ag/AgBr | Ag/AgI | Ag/AgCl | Ag/AgBr | Ag/AgI |
| Initial Conc | 6.423 | 6.423 | 6.423 | 1 | 1 | 1 |
| t0 | 3.49 | 5.054 | 7.295 | 0.54336 | 0.78686 | 1.135762 |
| t1 | 2.692 | 2.541 | 3.643 | 0.419119 | 0.39561 | 0.56718 |
| t2 | 1.769 | 1.622 | 2.992 | 0.275416 | 0.25253 | 0.465826 |
| t3 | 1.434 | 1.288 | 2.279 | 0.22326 | 0.200529 | 0.354819 |
| t4 | 1.062 | 1.101 | 1.872 | 0.165343 | 0.171415 | 0.291453 |
| t5 | 1.955 | 0.866 | 1.373 | 0.304375 | 0.134828 | 0.213763 |
|  |  |  |  |  |  |  |
| **UV Light Photocatalysis** | | |  |  |  |  |
|  | Concentration | | | Normalized | | |
| Time | Ag/AgCl | Ag/AgBr | Ag/AgI | Ag/AgCl | Ag/AgBr | Ag/AgI |
| Initial Conc | 7.966 | 5.625 | 5.625 | 1 | 1 | 1 |
| t0 | 7.021 | 4.845 | 5.357 | 0.881371 | 0.861333 | 0.952356 |
| t1 | 1.228 | 3.426 | 4.441 | 0.154155 | 0.609067 | 0.789511 |
| t2 | 2.711 | 2.481 | 3.426 | 0.340321 | 0.441067 | 0.609067 |
| t3 | 1.456 | 1.641 | 2.993 | 0.182777 | 0.291733 | 0.532089 |
| t4 | 1.213 | 1.992 | 2.472 | 0.152272 | 0.354133 | 0.439467 |
| t5 | 1.072 | 1.137 | 1.915 | 0.134572 | 0.202133 | 0.340444 |

|  | **Average UV Light Photocatalysis** | | | |  |  |  |  |  |  |
| --- | --- | --- | --- | --- | --- | --- | --- | --- | --- | --- |
|  |  | Average Concentration | | | Average Normalized | | | Standard Deviation (SD) | | |
|  | Time | Ag/AgCl | Ag/AgBr | Ag/AgI | Ag/AgCl | Ag/AgBr | Ag/AgI | Ag/AgCl | Ag/AgBr | Ag/AgI |
| -1 | Initial Conc | 7.1945 | 6.024 | 6.024 | 1 | 1 | 1 | 0 | 0 | 0 |
| 0 | t0 | 5.2555 | 4.9495 | 6.326 | 0.730489 | 0.82163 | 1.050133 | 0.23901 | 0.052661 | 0.129688 |
| 1 | t1 | 1.96 | 2.9835 | 4.042 | 0.27243 | 0.495269 | 0.670983 | 0.187358 | 0.150937 | 0.157212 |
| 2 | t2 | 2.24 | 2.0515 | 3.209 | 0.311349 | 0.340554 | 0.532703 | 0.045895 | 0.133316 | 0.101286 |
| 3 | t3 | 1.445 | 1.4645 | 2.636 | 0.200848 | 0.243111 | 0.437583 | 0.028626 | 0.064491 | 0.125349 |
| 4 | t4 | 1.1375 | 1.5465 | 2.172 | 0.158107 | 0.256723 | 0.360558 | 0.009243 | 0.129201 | 0.104662 |
| 5 | t5 | 1.5135 | 1.0015 | 1.644 | 0.210369 | 0.166252 | 0.272908 | 0.120069 | 0.047592 | 0.089577 |

| **VisiblePhotocatalysis** | |  |  |  |  |  |
| --- | --- | --- | --- | --- | --- | --- |
|  | Concentration | | | Normalized | | |
| Time | Ag/AgCl | Ag/AgBr | Ag/AgI | Ag/AgCl | Ag/AgBr | Ag/AgI |
| Initial Conc | 6.768 | 6.768 | 6.768 | 1 | 1 | 1 |
| t0 | 5.112 | 4.912 | 5.044 | 0.755319 | 0.725768 | 0.745272 |
| t1 | 4.589 | 3.34 | 5.5 | 0.678044 | 0.493499 | 0.812648 |
| t2 | 3.345 | 1.664 | 4.914 | 0.494238 | 0.245863 | 0.726064 |
| t3 | 3.503 | 1.351 | 4.344 | 0.517583 | 0.199616 | 0.641844 |
| t4 | 2.977 | 1.09 | 3.962 | 0.439864 | 0.161052 | 0.585402 |
| t5 | 2.177 | 0 | 3.582 | 0.321661 | 0 | 0.529255 |
|  |  |  |  |  |  |  |
| **Visible Photocatalysis** | | |  |  |  |  |
|  | Concentration | | | Normalized | | |
| Time | Ag/AgCl | Ag/AgBr | Ag/AgI | Ag/AgCl | Ag/AgBr | Ag/AgI |
| Initial Conc | 7.001 | 7.001 | 7.001 | 1 | 1 | 1 |
| t0 | 6.201 | 6.384 | 6.359 | 0.885731 | 0.91187 | 0.908299 |
| t1 | 5.346 | 3.523 | 6.562 | 0.763605 | 0.503214 | 0.937295 |
| t2 | 3.909 | 2.636 | 5.798 | 0.558349 | 0.376518 | 0.828167 |
| t3 | 3.115 | 2.106 | 5.334 | 0.444936 | 0.300814 | 0.761891 |
| t4 | 2.326 | 1.584 | 4.865 | 0.332238 | 0.226253 | 0.694901 |
| t5 | 1.57 | 1.485 | 4.954 | 0.224254 | 0.212113 | 0.707613 |

|  | **Average Visible Light Photocatalysis** | | | |  |  |  |  |  |  |
| --- | --- | --- | --- | --- | --- | --- | --- | --- | --- | --- |
|  |  | Average Concentration | | | Average Normalized | | | Standard Deviation (SD) | | |
|  | Time | Ag/AgCl | Ag/AgBr | Ag/AgI | Ag/AgCl | Ag/AgBr | Ag/AgI | Ag/AgCl | Ag/AgBr | Ag/AgI |
| -1 | Initial Conc | 6.8845 | 6.8845 | 6.8845 | 1 | 1 | 1 | 0 | 0 | 0 |
| 0 | t0 | 5.6565 | 5.648 | 5.7015 | 0.820525 | 0.818819 | 0.826785 | 0.092215 | 0.131594 | 0.115277 |
| 1 | t1 | 4.9675 | 3.4315 | 6.031 | 0.720824 | 0.498356 | 0.874971 | 0.060501 | 0.00687 | 0.088139 |
| 2 | t2 | 3.627 | 2.15 | 5.356 | 0.526293 | 0.31119 | 0.777116 | 0.045333 | 0.092387 | 0.072198 |
| 3 | t3 | 3.309 | 1.7285 | 4.839 | 0.48126 | 0.250215 | 0.701868 | 0.051369 | 0.071558 | 0.084886 |
| 4 | t4 | 2.6515 | 1.337 | 4.4135 | 0.386051 | 0.193653 | 0.640151 | 0.076103 | 0.046104 | 0.077427 |
| 5 | t5 | 1.8735 | 0.7425 | 4.268 | 0.272957 | 0.106056 | 0.618434 | 0.068877 | 0.149986 | 0.126118 |
